# Supplementary material for: Chromothripsis during telomere crisis is independent of NHEJ, and consistent with a replicative origin
Source: Genome Res. 2019 May;29(5):737–49. doi: 10.1101/gr.240705.118 (PMC6499312; doi:10.1101/gr.240705.118)
Supplement: Supplemental Material [file supp_gr.240705.118_Supplemental_file_1.zip › contigs/annotated_contigs/DB113/contig.2.DB113_length_721_mean_cov_15.0568654646.docx]

**DB113_length_721_mean_cov_15.0568654646**

```
GACCAACATAGCAAAACCCACTCCCTCCTTCATGA|GTAAAATAAAAATAATATAATTAATAAAAATACGAATAAGAACGCTTGGCCGG
                                    >chr10:12315738-12316019 + E=5e-150
GTGTGGTGGCTCACACCTGTAATCCCAGCACTTTGGGAGGCTGAGGCGGGTGGATCACAAGGTCAAGAGATCGAGACCATCCTGGCCAA

CATGGTGAAAACCCATCTCTACTAAAAATACAAAAATTAGCTGGACGTGGTGGGAGCCTGTAGTCCCAGCTACTTGGGAGGCTGAGGCA

AGAGAATCGCTTGAACCCTGGAGGCAGAGATTGCAATGAGCCGAGT|T|TTTTATCACCTGAAAAAGAAACCCCATACAAATTAACAGT
                                               >chr10:13281482-13281901 + E=1e-224
CACTCCTCATTTTTCCTAACCTCAAAGCCTTAGACAATCACTCTTCTTTGGTTTGTCTCTGGGGATTGGCCTATTCTAGACATTTCATG

TAAATAGAATCCTAAAATCTGTGGCCTTTTGTGACTGGTTTTCTTTCACTCAGCATAATGTTTTCAAGGCACATCCAGTTGCAGCATGC

ATCCATACCGCATCTTTTTTTTTTTTTGAGATGGAGTTTTCCTCTTGTCACCCAGGCTGGAGTGCAATGGCACAAGCTTGGCTCACTGA

AACCTCCGCCTCCTGGATTTAAGCAATTCTCCTGCCTCAGCCTCCTGAGTAGCTGGGACTACAGGCACCCGCCACCAAGCCTGGCTAAT

TTTTGTATATTT
```
